# The imaging conundrum of hepatic lymphoma revisited

**DOI:** 10.1007/s13244-015-0437-6

**Published:** 2015-10-06

**Authors:** S. Rajesh, Kalpana Bansal, Binit Sureka, Yashwant Patidar, Chhagan Bihari, Ankur Arora

**Affiliations:** Department of Radiology, Institute of Liver and Biliary Sciences, D-1, Vasant Kunj, New Delhi, 110070 India; Department of Pathology, Institute of Liver & Biliary Sciences, D-1, Vasant Kunj, New Delhi, 110070 India

**Keywords:** Hepatic lymphoma, Hepatitis-C, Periportal, Rim-enhancement, Scar

## Abstract

**Abstract:**

The imaging manifestations of hepatic lymphoma, both in its primary and secondary form, are extremely variable and overlap with a number of other more common hepatic diseases. However, in the appropriate clinical context, combining the imaging and laboratory features can aid in making the correct diagnosis. Since the management and prognosis of lymphomas are significantly different from other malignancies, early diagnosis and prompt commencement of therapy is of paramount importance. The various morphological appearances of hepatic lymphoma on imaging have been described here along with their possible differentials.

***Teaching points*:**

• *Primary hepatic lymphoma is extremely rare*.

• *Secondary liver involvement occurs in 50 % of patients with non-Hodgkin lymphoma*.

• *The imaging manifestations of hepatic lymphoma are largely non-specific*.

• *Some imaging features may be helpful in the appropriate clinical setting*.

• *Management and prognosis of lymphoma is significantly different from other malignancies*.

## Introduction

Secondary involvement of the liver can occur in up to 50 % of patients with non-Hodgkin lymphoma and in around 20 % of patients with Hodgkin disease [1, 2]. However, primary hepatic lymphoma (PHL) is rare, representing <1 % of all non-Hodgkin’s lymphoma [3, 4]. The past two decades have seen an increase in the number of reported cases of PHL, especially in the setting of immunosuppression [5–15]. Although the imaging manifestations of hepatic lymphomas are largely non-specific, there are some features that may serve as a useful diagnostic clue in the apppropriate clinical setting. Since multiagent chemotherapy is the mainstay of treatment in secondary hepatic lymphoma (SHL) with surgery being reserved for cases of PHL with resectable disease, early and accurate diagnosis can alter the therapeutic protocol and obviate the need for unnecessary radical surgery. In this article, we attempt to classify hepatic lymphomas based on the different morphological patterns seen on imaging at presentation and discuss the various other more common hepatic lesions that need to be differentiated from lymphoma.

## Primary hepatic lymphoma

PHL is defined as lymphoma that is confined to the liver and perihepatic lymph nodes, without evidence of involvement of other visceral organs, distant lymph nodes or bone marrow for at least 6-months after the onset of hepatic disease [16]. PHL should be differentiated from lymphoma that secondarily affects the liver because the management and prognoses differ significantly. Although first described way back in 1965 by Ata and Kamal [17], only 100 odd cases of PHL have since been reported in English-language medical literature [8, 18]. This is because host factors can make the liver a poor environment for the development of malignant lymphoma [8].

PHL is usually diagnosed in the 4th-5th decade and shows a male predominance [14]. It is commonly associated with viral hepatitis B and C, Epstein-Barr virus, and human immunodeficiency virus [14]. Hepatitis-C viral infection is particularly common, being found in 20–60 % of patients with PHL [19, 20]. Clinical symptoms including right upper abdominal pain, weight loss, and fever can be seen in up to 50 % of cases. The majority of the cases of PHL are of B-cell lineage, and diffuse large B-cell lymphoma is the most commonly detected histological subtype [14].

## Secondary hepatic lymphoma

Secondary hepatic involvement by lymphoma is relatively common and indicates advanced disease. Patients usually present with systemic symptoms of fever, night sweats, and weight loss. Hepatosplenomegaly and generalized lymphadenopathy can be commonly found on systemic examination [21]. Mild elevation of serum transaminases and moderate elevation of alkaline phosphatase (ALP) can occur due to tumour infiltration or extrahepatic bile duct obstruction [21]. Differences between primary and secondary hepatic lymphoma have been summarized in Table 1.

**Table 1:** Clinical and radiological manifestations of primary vs. secondary hepatic lymphoma

		Primary hepatic lymphoma	Secondary hepatic lymphoma
Clinical	Definition	Lymphoma confined to the liver and perihepatic nodal sites (without distant involvement) at time of presentation	Lymphoma that secondarily affects the liver
	Incidence	1 % of non-Hodgkin lymphoma	50 % of non-Hodgkin lymphoma 20 % Hodgkin lymphoma
	Associations	Epstein-Barr virus, hepatitis B and C infection, HIV infection and cirrhosis	-
	Presenting complaints	Right upper quadrant pain, jaundice (10-20 %)	Systemic symptoms (B symptoms) such as fever, night sweats, weight loss
	Clinical examination	Hepatomegaly/ palpable liver	Systemic lymphadenopathy, hepatosplenomegaly
	Bone marrow involvement/ Leukemic blood profile	Absent (for at least for 6 months after first presentation)	Present
Radiological	Lesion distribution	Solitary discrete lesion (60 %), multiple lesions (35-40 %)	Multifocal lesions or diffuse infiltration (90 %) Miliary shadows (10 %)
	Dominant mass(es)	Typically present	Usually absent
	Morphology	Heterogeneous	Homogeneous
	Enhancement	Heterogeneous	Heterogeneous
	Splenic lesions	Absent	Present (30-40 %)
	Supra-and/or infra-diaphragmatic lymphadenopathy	Absent	Present

## Other forms of lymphoma

*Hepatosplenic T-cell lymphoma* is a rare and poorly recognized entity characterized by neoplastic proliferation of T-cells in hepatic sinusoids and splenic red pulp without lymphadenopathy and bone marrow involvement [22–26]. More recently, hepatic involvement by the *intravascular variety of lymphoma* has also been described caused by neoplastic proliferation in the lumen of small- to medium-sized hepatic vessels [27, 28]. However, due to paucity of literature on these forms of lymphoma, no specific imaging features have been recognized. The reported cases of hepatosplenic lymphoma usually presented with features of hepato-splenomegaly or cirrhosis without evidence of any focal lesions [24–26], while the only report on the imaging manifestations of hepatic intravascular lymphoma described non-specific focal liver lesions and areas of uneven perfusion [27].

## Imaging features

Lymphomatous involvement of liver can manifest on imaging as solitary or multiple nodular lesions, diffuse infiltration, or as a periportal soft tissue mass [15, 28, 29]. A combination of infiltrating and nodular patterns has also been described.

**Fig. 1:**
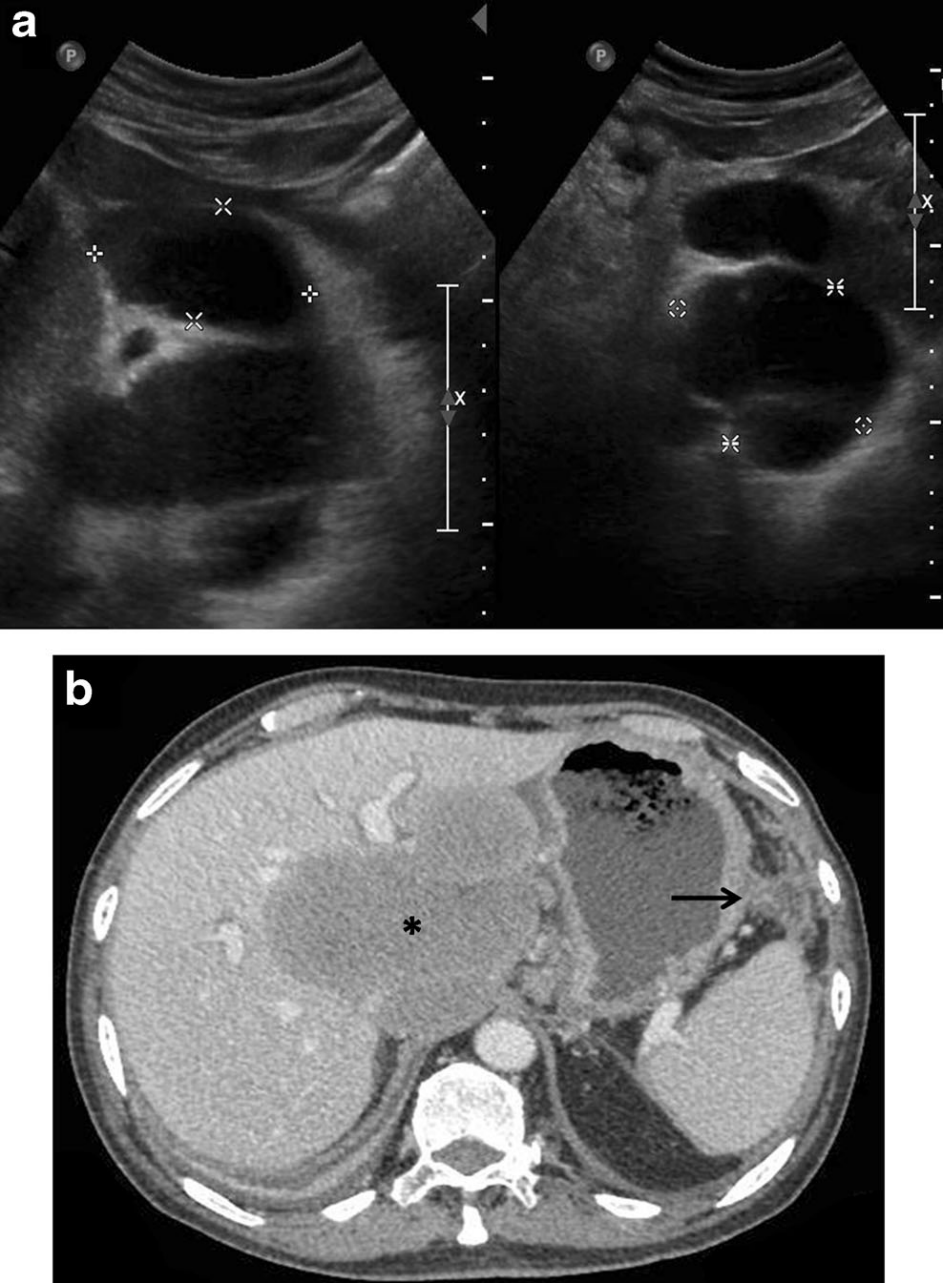
Incidentally detected PHL in a 76-year-old man with acute pancreatitis. **a** Gray-scale US images demonstrating a lobulated anechoic lesion (partly marked by calipers) in the gastrohepatic ligament abutting the liver. This was reported as suspicious for pancreatic pseudocysts in view of the clinical history. **b** Axial CECT image of the same patient showing a homogeneous, partially exophytic, hypoenhancing mass (asterisk) replacing the caudate lobe of the liver and involving the adjoining hepatic parenchyma. Biopsy from the liver lesion revealed findings consistent with diffuse large B-cell lymphoma. Sequel of acute pancreatitis can be seen as ill-defined soft tissue stranding along the greater curvature of the stomach (arrow)

**Fig. 2:**
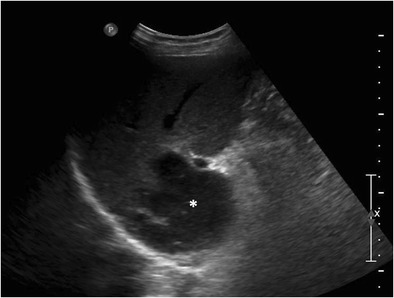
PHL in a 42-year-old man with right upper abdominal pain. Gray-scale US image showing a partially exophytic hypoechoic lesion (asterisk) in the right lobe of liver suspicious for abscess. The patient underwent diagnostic aspiration followed by biopsy from the lesion, which revealed findings consistent with diffuse large B-cell lymphoma

1. Nodular (mass-forming) pattern
  - Solitary discrete lesion
    This is the most common presentation of PHL on imaging, seen in approximately 60 % of cases [12–14]. SHL, on the other hand, manifests in this manner in only about 10 % of cases. On ultrasound (US), the lesion is usually well defined and appears markedly hypoechoic or anechoic, occasionally mimicking a cyst [10, 29] (Fig. 1). This is because lymphoma is a homogeneous tumour and generates very few internal reflections. However, absence of posterior acoustic enhancement can be a useful pointer towards the solid nature of the lymphomatous lesion [29]. Perihepatic regional lymph nodal involvement is commonly seen. Instances of hepatic lymphoma being confused with an abscess on ultrasound (US) have also been reported [30–32]. The hypoechoic appearance of lymphoma along with the presence of fever and other systemic symptoms in these patients may make it extremely difficult to distinguish lymphoma from an abscess (Fig. 2). Presence of mobile internal echoes, septations, and heterogeneity favours the diagnosis of abscess. In addition, reactive right pleural effusion, ascites, and bowel abnormalities (caecal wall thickening in cases of amoebic infection) can be found in patients with hepatic abscess (Table 2).

**Table 2:** Differentials of various morphological types of hepatic lymphoma

Morphological Type	Differentials	Distinguishing feature(s)
Solitary discrete lesion	Cyst	- Posterior acoustic enhancement on US
	Abscess	- Mobile internal echoes, septations and heterogeneity on US - Reactive right pleural effusion, ascites and caecal wall thickening - Presence of surrounding edema and pylephlebitis - Absence of vessel penetration sign
	Hepatocellular carcinoma (HCC)	- Arterial phase enhancement - Venous invasion/thrombosis - Raised alpha-fetoprotein levels
	Focal nodular hyperplasia	- Homogeneous enhancement
	Intrahepatic cholangiocarcinoma	- Capsular retraction - Mass effect in the form of biliary dilatation and vascular displacement - Increased CA-19.9 levels
Multifocal lesions	Tuberculosis	- Necrotic rim-enhancing lymph nodes - Lung parenchymal involvement
	Sarcoidosis	-T2-hypointense nodules - Pulmonary involvement - Hilar lymphadenopathy
	Fungal microabscesses	- Perilesional edema - History of immunosuppression - High-grade fever with increased white blood cell count
	Septic emboli	- Known source of infection - Clinical features of sepsis
	Metastases	- Known primary malignancy
Diffuse infiltration	Infiltrative HCC	- Venous invasion/thrombosis - Markedly raised alpha-fetoprotein levels - Often non-avid on FDG PET-CT
	Acute viral hepatitis	- Echogenic periportal cuffing on US - Normal or mildly elevated LDH levels - Significant elevation of transaminases
Periportal mass	Periportal edema	- Echogenic on US - History of trauma, surgery
	Biliary dilatation	- Anechoic tubular structures on US - Absence of complete encasement of portal venous radicals
    The lesion is usually homogeneously hypodense on non-contrast-enhanced computed tomographic (CT) scan and has soft tissue attenuation. Areas of necrosis and haemorrhage may occasionally be seen. However, calcification is rare in the absence of treatment [29].
    Upon administration of intravenous contrast agent, two different types of enhancement patterns have been recognized in lymphomatous nodules. These enhancement characteristics also apply to the multinodular pattern of lymphomatous liver infiltration described later:
    Majority of the lesions demonstrate minimal to no enhancement on all the phases [12, 29]. Enhancement, when present, is characteristically less than the surrounding hepatic parenchyma (Fig. 3). However, multiple vascular channels can often be seen coursing through the lesion. This has been referred to as the “vessel penetration sign” (Fig. 4). In addition to this, the absence of any significant surrounding oedema and pylephlebitis can be used to differentiate lymphoma from hepatic abscess. Since PHL is commonly associated with Hepatitis-C and B viral infections, one may come across lymphomatous hepatic lesion on a background of cirrhotic liver (Fig. 4). In these instances, it may be difficult to differentiate PHL from hepatocellular carcinoma (HCC). However, in typical cases, absence of significant arterial phase enhancement, vessel penetration sign, and absence of vascular thrombosis are useful clues to diagnose lymphoma (Table 2). In addition, laboratory investigations like tumour markers can also be used to support the imaging diagnosis.

**Fig. 3:**
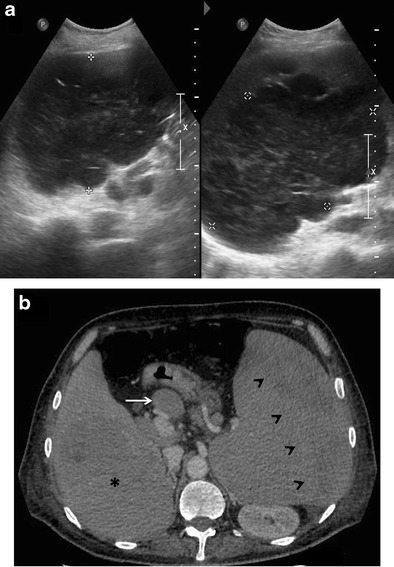
SHL in a 38-year-old man with fever and significant weight loss. **a** Gray scale US image showing a large lobulated hypoechoic lesion (marked by calipers) in the right lobe of liver. **b** Axial CECT image demonstrating a subtle well-defined hypoenhancing lesion (asterisk) in liver with perihepatic lymph nodes (arrow), splenomegaly, and altered enhancement of the splenic parenchyma (arrowheads)

**Fig. 4:**
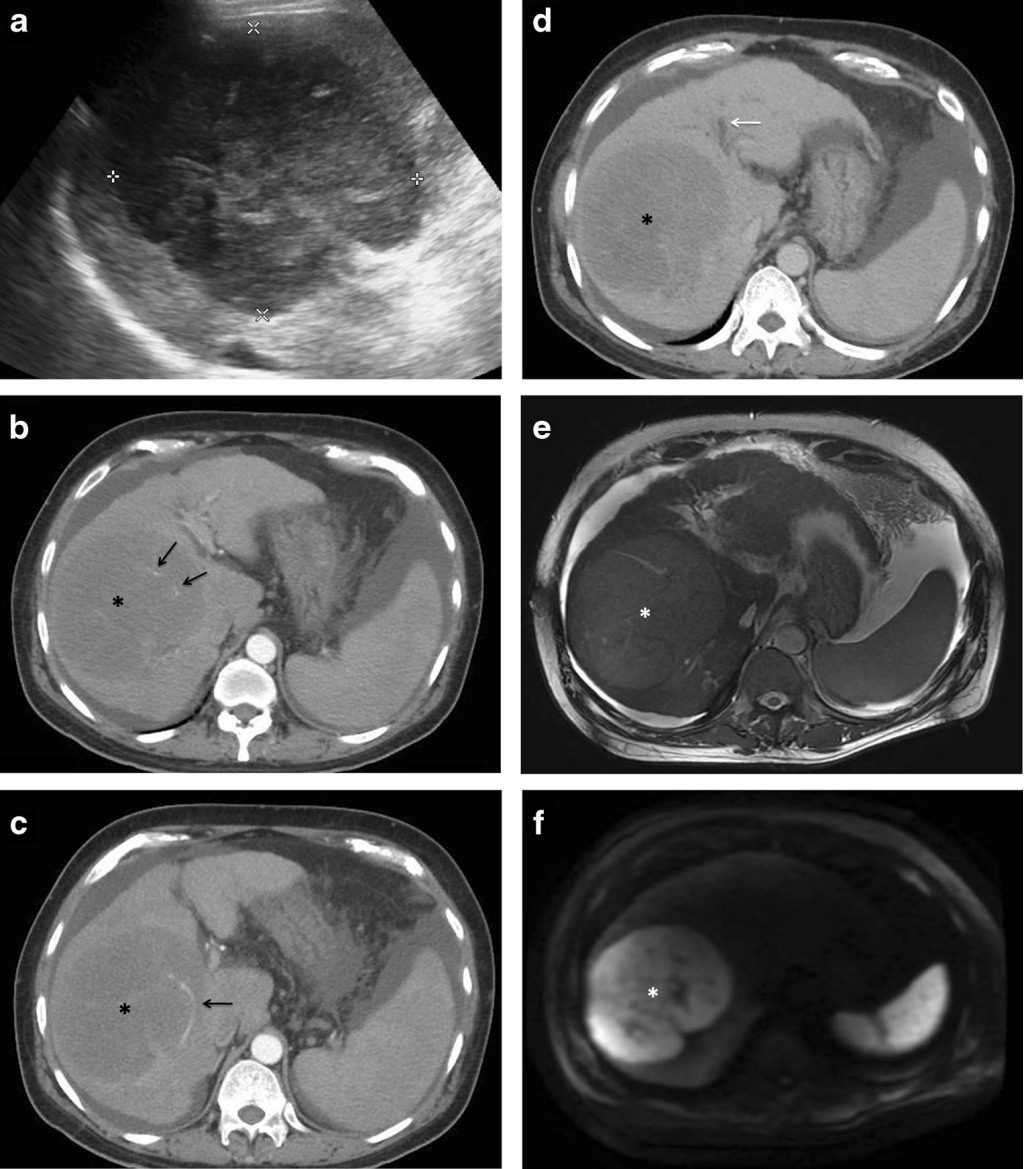
Primary hepatic non-Hodgkin lymphoma in a 55-year-old man with jaundice. **a** Gray-scale US image showing a large, well-defined, markedly hypoechoic lesion in the right lobe of liver (marked by calipers). CECT images (**b**-**d**) demonstrating a hypoenhancing lesion (asterisks) with arterial channels coursing through it (arrows in **b** and **c**). Changes of liver cirrhosis and ascites can also be seen with biliary dilatation (arrow in **d**). MR images reveal a homogeneous, mildly T2-hyperintense, diffusion restricting lesion (asterisk in **e** and **f**)
      Occasionally, a central “scar” can be seen in this type of hepatic lymphoma, mimicking focal nodular hyperplasia [33] (Fig. 5). However, poor enhancement of the lesion on all the contrast-enhanced phases allows one to confidently differentiate between the two entities.

**Fig. 5:**
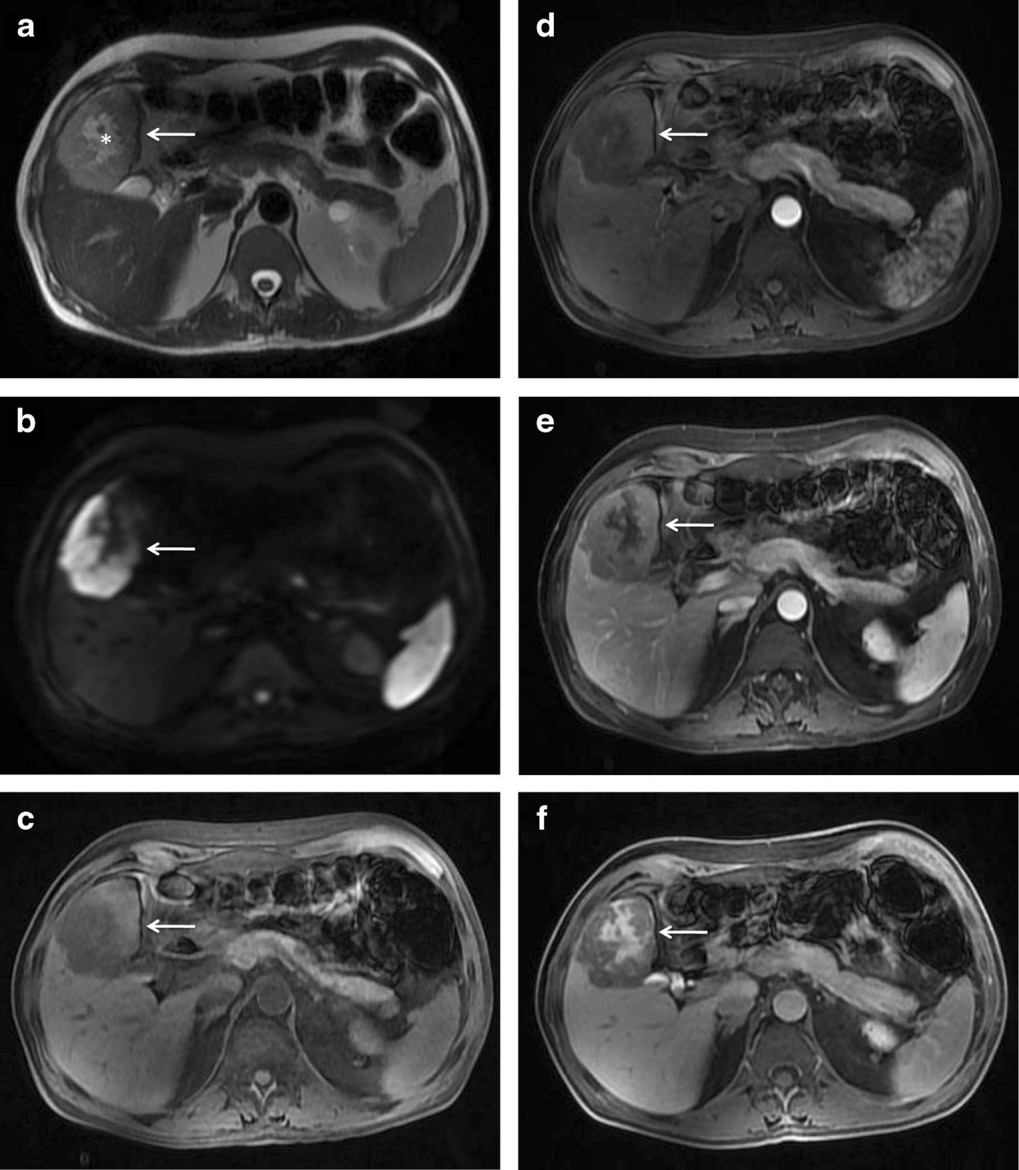
Diffuse large B-cell lymphoma with secondary liver involvement in a 57-year-old man. (**a**) Axial T2-weighted MR image demonstrating a well-defined, lobulated, mildly hyperintense lesion (arrow) with a more hyperintense central “scar.” It shows peripheral diffusion restriction (arrow in **b**). The lesion is hypointense on T1-weighted image (arrow in **c**). On contrast-enhanced scan the lesion shows mild progressive enhancement in the arterial and venous phase images (arrows in **d** and **e**), which is less than the surrounding liver parenchyma while the scar remains predominantly non-enhancing. On the 1-hr delayed hepatobiliary phase, the lesion remains hypointense to the surrounding liver parenchyma while there is retention of contrast within the scar
    The other pattern is that of enhancement of the rim of the lesion with a central non-enhancing area giving a target-like appearance to the lesion [29, 34]. Due to the well-defined, lobulated outline of these lesions and the predominantly peripheral enhancement, which persists even on the equilibrium phase images, they can be misdiagnosed as intrahepatic cholangiocarcinoma on imaging (Fig. 6). But, unlike a cholangiocarcinoma, capsular retraction and mass effect in the form of biliary dilatation and vascular displacement is typically absent in hepatic lymphoma. Also, CA-19.9 levels are often significantly increased in cholangiocarcinoma.

**Fig. 6:**
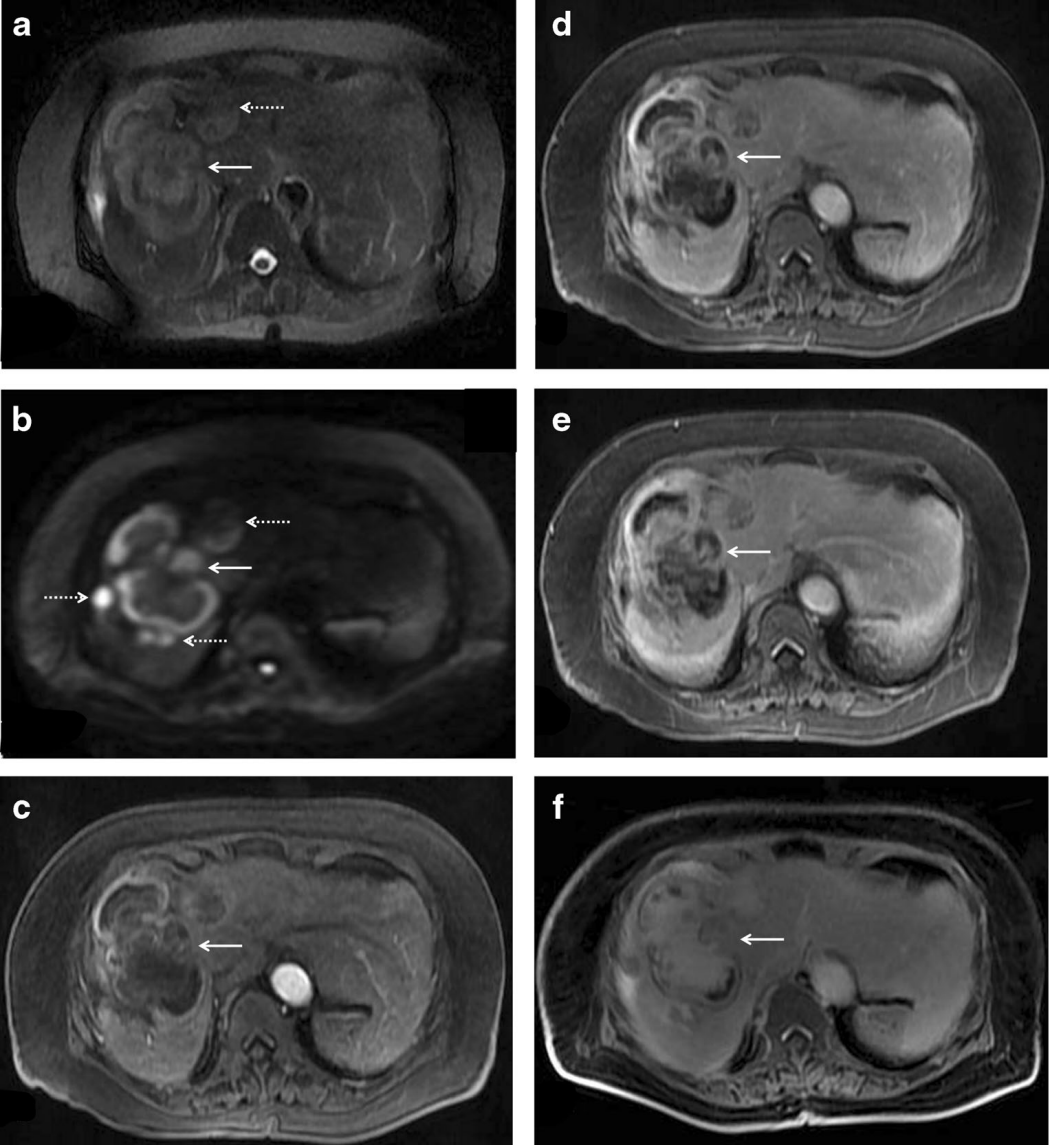
Rim-enhancing masses in a 65-year-old woman with PHL. Axial T2-weighted fat-saturated MR image showing a well-defined lobulated hyperintense lesion (solid arrow in **a**) with peripheral diffusion restriction (**b**). Multiple other similar but smaller nodules (interrupted arrows) are also seen. Post contrast administration, rim-enhancement of the dominant lesion is seen on the arterial phase image (**c**) with partial progressive centripetal fill-in of contrast on the subsequent portal venous (**d**) and equilibrium phase images (**e**). On the 1-hr delayed hepatobiliary phase, the central portion of the tumor shows accumulation of contrast. The other nodules also demonstrate similar enhancement pattern
    On magnetic resonance (MR) imaging, the lesion tends to be homogeneously hypo to isointense on T1-weighted images and hyperintense on T2-weighted images [29, 35]. Signal intensity on T2-weighted images may be heterogeneous due to foci of haemorrhage and necrosis [12]. T2-hypointense tumours with peripheral rim of hyperintensity have also been reported. The increased signal around the lesion has been attributed to the inflammatory response elicited by the lymphomatous lesion and resultant surrounding oedema [12]. Similarly, target-like lesions showing a T2-hyperintense rim with a slightly more hyperintense core can also be seen (Fig. 6). Enhancement patterns on MRI are similar to that seen on CT. However, when hepatobiliary specific MRI contrast media are used, retention of contrast by the centre of the lesion can be seen on the delayed hepatobiliary phase (Fig. 6).
    The highly cellular nature of lymphoma typically results in restricted diffusion and whole-body diffusion-weighted imaging has been suggested to be as sensitive as FDG-PET/CT in staging of lymphoma [36]. Occasionally a rim of diffusion restriction may also be seen (Fig. 6). Moreover, quantification of diffusion effects of the tumour using mean apparent diffusion coefficient (ADC) values also helps in characterization of these lesions. Using b values of 100, 500, and 750 s/mm2, Badawy et al. [37] demonstrated that hepatic lymphomas showed mean ADC values of 1.37, 1.20 and 1.18, respectively, which were significantly lower than that for benign cystic hepatic lesions and benign tumours like hemangioma, adenoma, and FNH, while being similar to malignant tumours like HCC. They concluded that using different b values can not only increase sensitivity for detection of additional liver lesions but also offers the possibility to characterize focal liver lesions into benign and malignant without the need for contrast agent administration by using ADC measurements.
    FDG-PET/CT is the imaging modality of choice for staging and response assessment of FDG-avid lymphomas and obviates the need for pretreatment bone marrow biopsy in patients with Hodgkin lymphoma and most cases of diffuse large B-cell lymphoma [38]. On FDG-PET/CT, the lesion typically demonstrates avid hypermetabolism (Fig. 7). The value of FDG-PET/ CT in addition to CT lies in the high lesion-to-background contrast and quantification of glucose metabolism (using measurement of standardized uptake value). Standardized uptake value may also be valuable as a biomarker in assessing the tumour grade, for guiding biopsy, and for determination of response to therapy in patients with lymphoma. Disadvantages of FDG-PET/CT, however, are exposure of the patient to ionizing radiation and relatively high cost. A number of studies have recently compared whole body DWI with FDG-PET/CT in staging of lymphoma at initial presentation and have found moderate to good interobserver agreement [36]. Since MRI does not entail ionizing radiation, allows cross-sectional imaging of the entire body, provides a higher soft-tissue contrast, and offers a wide variety of anatomical and functional sequences, it may be used as an alternative to FDG-PET/CT in this regard [36]. However, there are still no recommendations on the routine use of MRI in this regard.

**Fig. 7:**
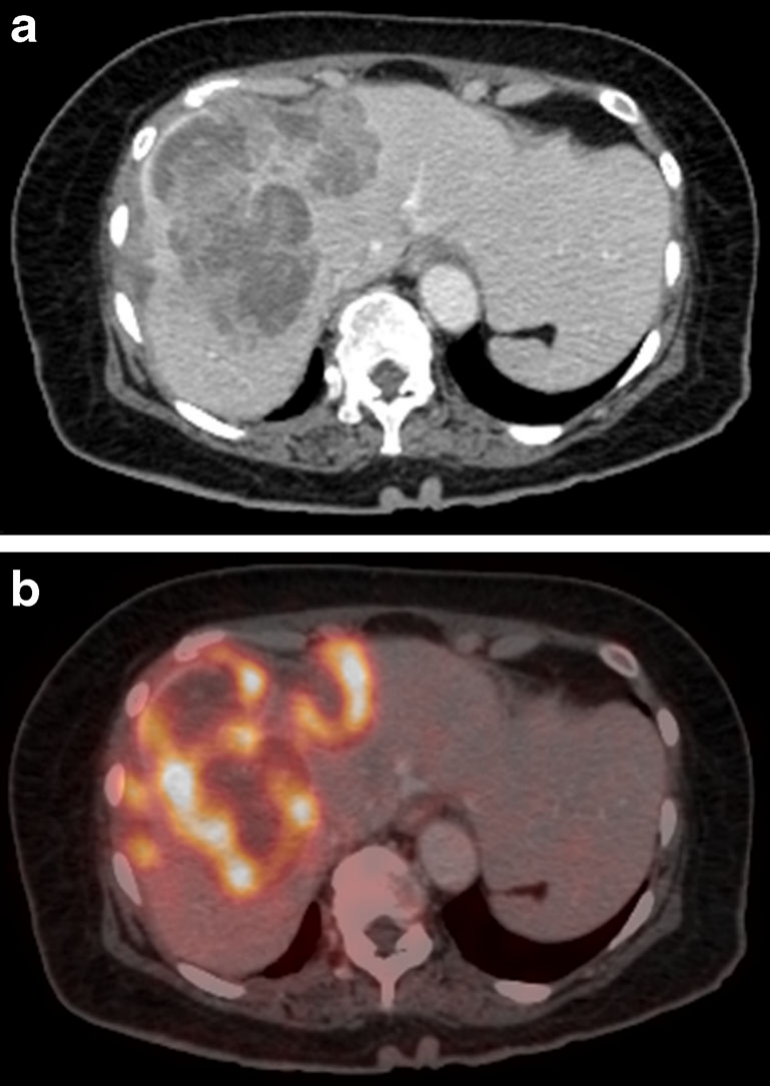
Axial contrast-enhanced CT (**a**) and corresponding FDG-PET (**b**) images of the same patient as in Fig. 6 showing avid FDG uptake within the predominantly hypoenhancing hepatic lesions (*Image courtesy: Dr. Ishita Sen, Dept. of Nuclear Medicine, Fortis Memorial Research Institute, Gurgaon*)
  - Multifocal lesions (with or without a dominant mass)
    Multiple discrete hepatic lesions of varying sizes have been reported at presentation in approximately 35- 40 % of cases of PHL [10, 12], although one of the lesions is typically dominant [29]. In contrast, multifocal lesions or diffuse infiltration is the most common pattern of liver involvement in SHL, seen in up to 90 % of cases. Dominant masses are usually absent in SHL. Also, untreated nodules in SHL, even when large, are usually homogeneous (Fig. 8), while the dominant masses in PHL are typically heterogeneous. Concomitant splenic lesions can be seen in 30-40 % of patients with SHL, which, along with widespread infra- or supradiaphragmatic lymphadenopathy and bone marrow infiltration facilitate correct diagnosis [29]. The nodules are markedly hypoechoic on US. They may also have a target appearance, with central echogenic and peripheral hypoechoic components (Fig. 9). As mentioned previously, the enhancement patterns of the individual lesions in multinodular lymphoma are essentially similar to those described above for the solitary mass-forming variety of lymphoma. Thus, the nodules are typically diffusely hypoenhancing compared to the surrounding hepatic parenchyma. Patchy enhancement or rim-enhancement of the nodules can be seen in up to 15 % of cases (Figs. 10 and 11).

**Fig. 8:**
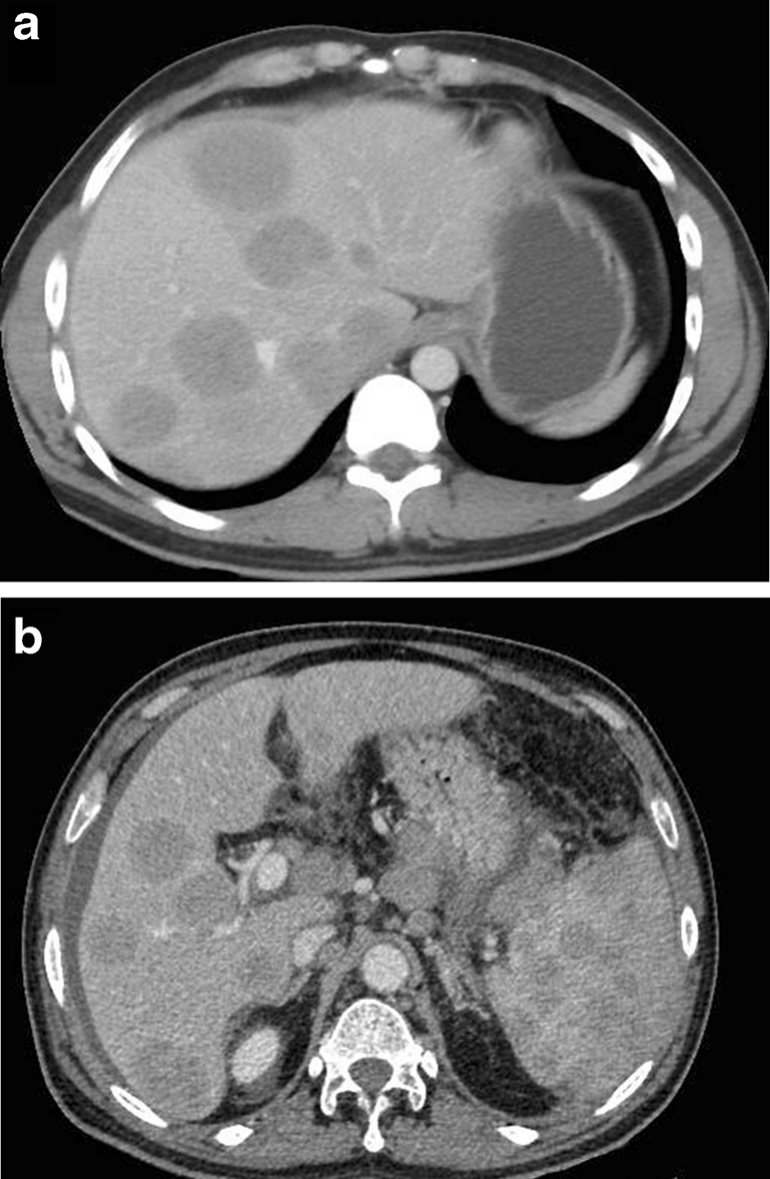
CT findings in SHL from two different patients. Axial CECT images demonstrating multiple homogeneous hypoenhancing hepatic lesions. Splenic lesions and perihepatic lymph nodes can also be seen in the image **b**

**Fig. 9:**
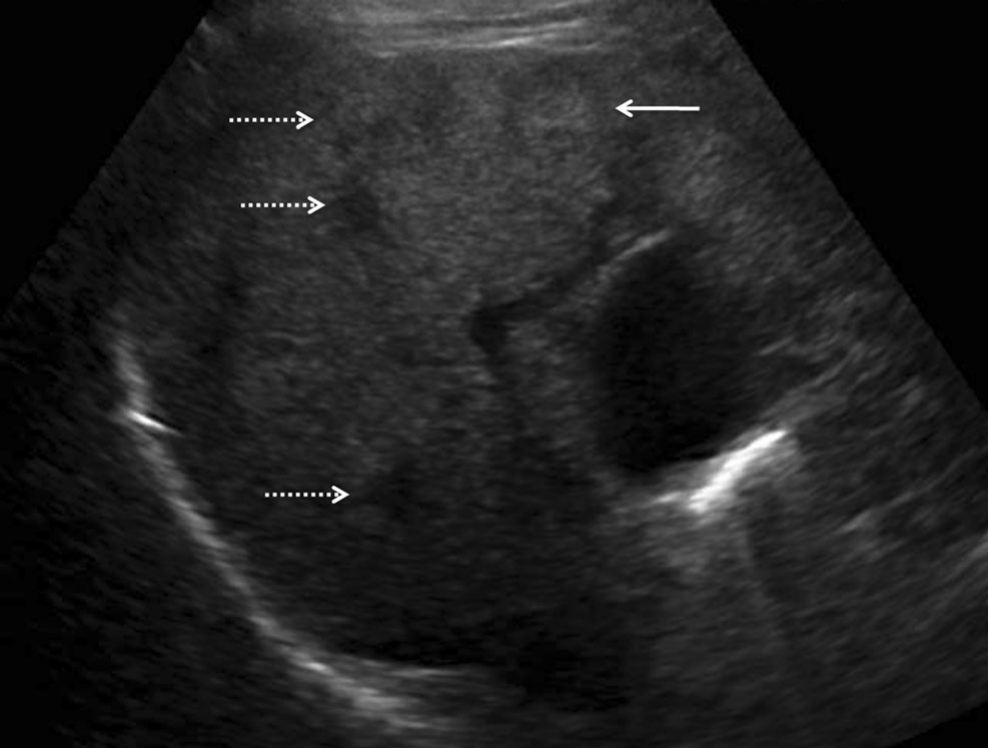
Secondary anaplastic large cell lymphoma of liver in a 48-year-old man. Gray-scale US image demonstrating a focal nodular lesion in liver (solid arrow), which shows a central echogenic component and a peripheral ill-defined hypoechoic ring (target-sign). Multiple other hypoechoic lesions can also be seen (interrupted arrows) in liver. This appearance cannot be distinguished from metastases on imaging, and tissue diagnosis is mandatory in the absence of any known primary malignancy

**Fig. 10:**
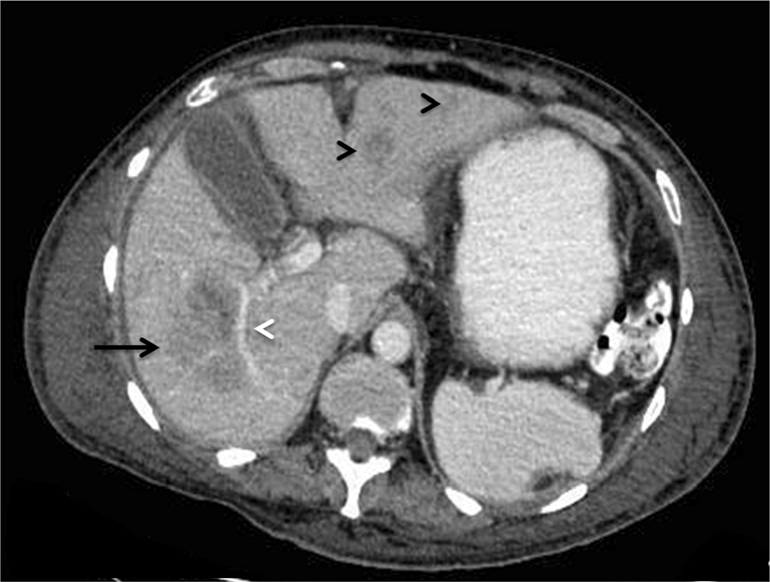
PHL manifesting as multifocal lesions in a 67-year-old male with fatigue and weight loss. Axial CECT image showing a hypoenhancing dominant lesion in the right lobe of liver (arrow) with vessel penetration sign (asterisk). Few other similar but smaller lesions are noted in the left lobe (arrowheads)

**Fig. 11:**
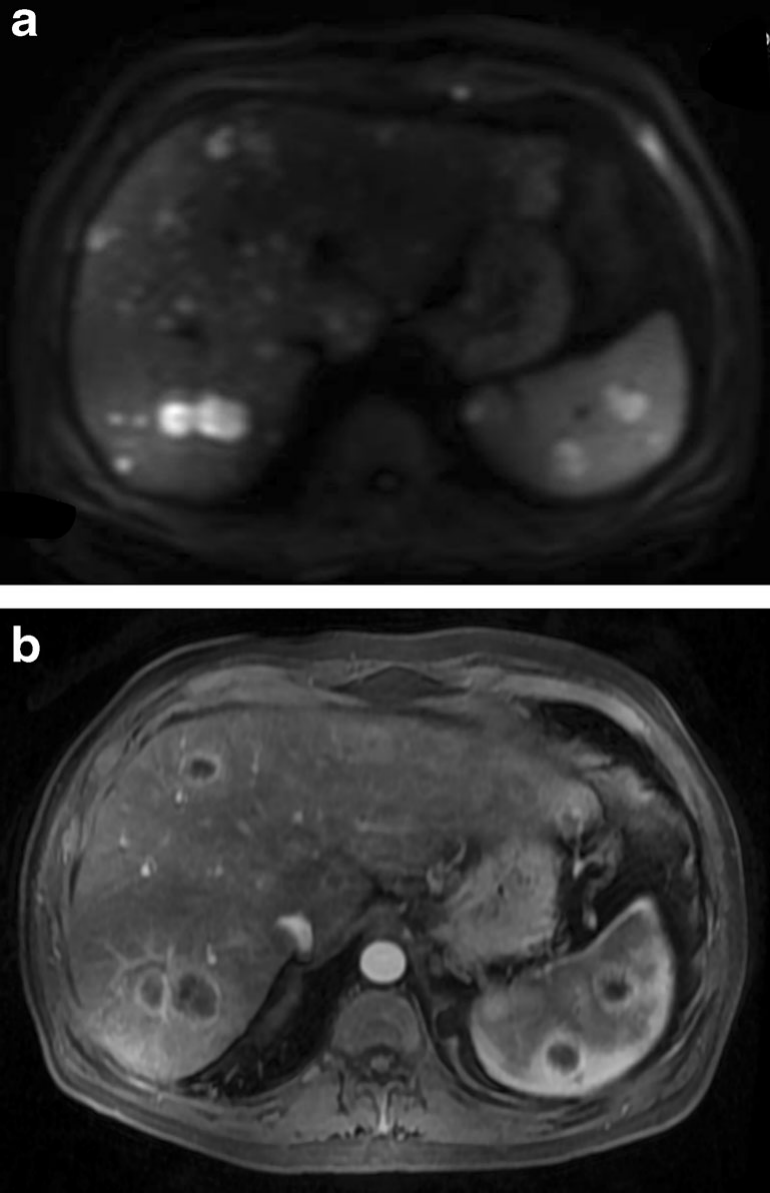
SHL in a 56-year-old man with fever and significant weight loss. **a** Multiple well-defined variable sized diffusion restricting nodules are seen involving both liver and spleen. **b** On post administrations of intravenous contrast, the nodules demonstrate rim-enhancement. This appearance can be difficult to differentiate from abscesses and disseminated granulomatous infections. Histopathological examination revealed this to be diffuse large B-cell lymphoma with bone marrow involvement
    On MR imaging, the nodules are usually hypo- or isointense on T1-weighted images and hyperintense on T2-weighted images, although T2-hypointense nodules mimicking hepatic sarcoidosis have been reported [12].
    The list of differentials for the multifocal pattern of presentation of hepatic lymphoma is long and includes disseminated granulomatous infections (tuberculosis and sarcoidosis), fungal microabscesses, septic emboli, and metastases. Of these, hepatic involvement caused by granulomatous diseases can be particularly challenging to differentiate from lymphoma, since they can also result in hypoenhancing hepato-splenic lesions with splenomegaly and extensive lymphadenopathy. In addition, systemic symptoms of fever and weight loss are also commonly present, further confounding the issue. Presence of necrotic rim-enhancing lymph nodes in tuberculosis and T2-hypointense nodules in sarcoidosis along with the characteristic pattern of lung involvement in these granulomatous infections and clinical inputs are often helpful in making a correct diagnosis [39], although such differentiation is not always achievable in practice.
    In the case of fungal microabscesses, patients commonly provide a history of immune-suppression and present with high-grade fever and a raised white blood cell count. Perilesional oedema can be demonstrated in fungal microabscesses, although this may not be appreciable in smaller lesions. Patients with septic emboli usually have a known source of infection. Similarly, metastatic disease should be considered when there is evidence of a known primary in the patient.
2. Diffuse infiltration
  Infiltration of tumour cells into the portal tracts as well as sinusoids is one of the most common patterns of hepatic involvement in cases of SHL. It is rare in PHL, but when present, it portends a poor prognosis [13]. There are no specific imaging features, and the only sign of hepatic involvement is diffuse enlargement of liver (Fig. 12). However, relying purely on physical examination or the measurement of the cranio-caudal span of liver on CT to detect enlargement can often produce fallacious results, especially on initial evaluation. This is because liver size is affected by body habitus, race, and a number of unrelated systemic diseases. Moreover, absence of universally accepted guidelines for the measurement of liver span on imaging affects interobserver agreement and reproducibility of these measurements. In these cases, assessment of liver volume using commercially available software (Myrian® XP Liver) provides an accurate and objective measure of liver enlargement, which can also be effectively reproduced on follow-up scans. FDG-PET/CT has also been shown to be helpful in these cases by demonstrating diffuse FDG uptake in the enlarged liver and spleen with systemic FDG-avid lymphadenopathy [40].

**Fig. 12:**
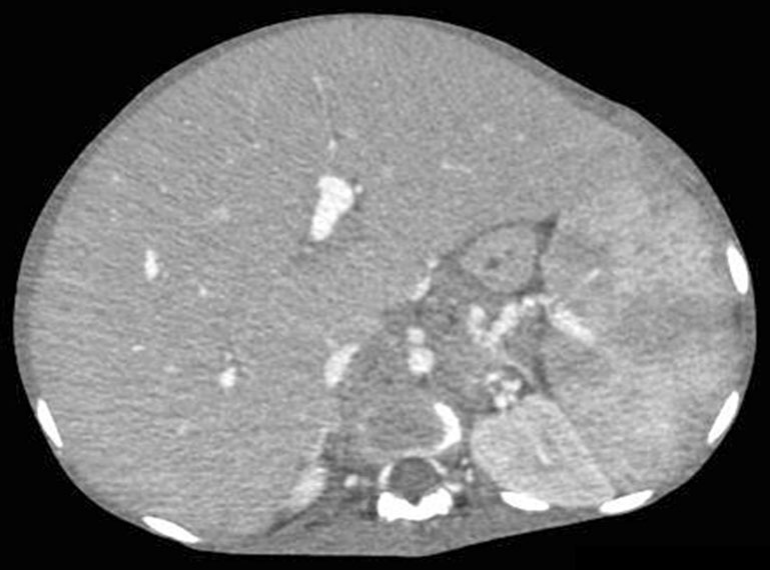
Infiltrating SHL in an 18-year-old female with abdominal pain and distension. Axial CECT image demonstrating diffuse enlargement of liver and spleen without any evidence of focal hepatosplenic lesions
  In cases of segmental hepatic infiltration, subtle T2-hyperintensity with diffusion restriction can occasionally be seen. Infiltrative HCC may be difficult to distinguish from this pattern of lymphomatous hepatic involvement, since both these entities can occur in cirrhotic livers and have similar imaging characteristics [41]. However, tumoral infiltration and attendant thrombosis of adjoining portal venous radicals is commonly seen in cases of infiltrative HCC, and the levels of alpha fetoprotein are usually markedly raised. Lymphoma, on the other hand, often presents with concomitant splenomegaly, lymphadenopathy below the level of the renal veins, and causes vascular encasement without thrombosis. Also, in general, hepatic lymphomas are avidly hypermetabolic at PET, while most HCCs are not.
  Not infrequently, patients with diffuse infiltrating lymphoma can present with fulminant hepatic failure due to extensive infiltration of sinusoids and hepatic vasculature by malignant cells resulting in diffuse hepatic necrosis [42]. On imaging, a diffusely oedematous gall bladder wall can be seen along with hepatosplenomegaly, ascites, and perihepatic lymphadenopathy mimicking acute viral hepatitis (AVH) [43]. Clinical deterioration in these patients often occurs rapidly and liver biopsy is usually required to make an accurate diagnosis although, in rare instances, even a biopsy might be inconclusive [44].
  A combination of infiltrating and nodular pattern of hepatic involvement can also be seen on imaging resulting in diffuse hepatic enlargement with multiple subtle hypoenhancing nodules scattered in the liver parenchyma (Fig. 13).

**Fig. 13:**
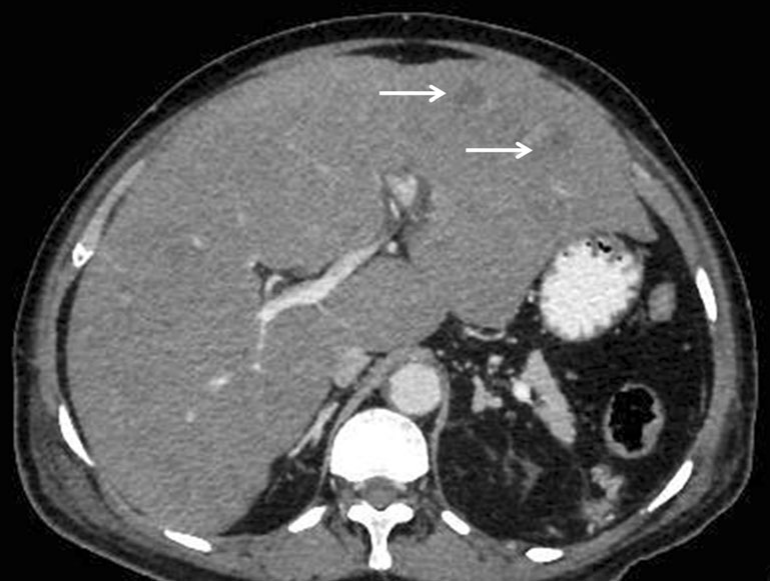
Mixed infiltrating and nodular variety of SHL in a 57-year-old man. Axial CECT image showing ill-defined hypoenhancing lesions in the left lobe of liver (arrows) on a background of hepatomegaly. Liver biopsy revealed lymphomatous infiltration of hepatic sinusoids
3. Periportal mass
  Periportal spread of lymphoma can be explained by the fact that lymphatic vessels along the portal vein and bile ducts are responsible for 80 % of hepatic lymph drainage [45]. This variant is being increasingly recognized and manifests in the form of periportal soft-tissue cuffing or ill-defined mass [46–48]. Both primary and secondary forms of hepatic lymphoma can present in this manner.
  When the involvement is limited to periportal soft tissue cuffing, this variety of hepatic lymphoma should be differentiated from the other causes of “periportal halo” like biliary dilatation, lymphedema (secondary to transplantation or portal lymphadenopathy), trauma, cardiac failure, and AVH. Out of these, AVH can be particularly difficult to differentiate from lymphoma on CT and MRI because in both these conditions, patients present with hepatosplenomegaly and periportal cuffing in the setting of deranged liver functions. However, US is helpful in such cases because periportal cuffing in lymphoma is usually hypoechoic while in AVH, it is echogenic (Fig. 14). Additionally, in AVH, direct and indirect bilirubin levels are usually elevated in roughly equal proportions and ALP and lactate dehydrogenase (LDH) levels are normal or only mildly elevated. In contrast, moderate elevation of ALP and LDH can occur in lymphoma due to tumour infiltration or extrahepatic bile duct obstruction with resultant direct hyperbilirubinemia. Also, in lymphoma, only a mild increase in serum transaminases is seen while AVH causes significant elevation in serum transaminase levels, often reaching up to 100 times their normal ranges. Viral markers can also used to differentiate between the two, although the coexistence of Hepatitis-B and C infection in patients with lymphoma limits its diagnostic utility. Bile duct dilatation can be differentiated from lymphoma by the complete encasement of the portal vessels by the tumour and its hypoechoic appearance as opposed to the anechoic biliary radicals. Other causes of periportal halo can usually be diagnosed on the basis of clinical history.

**Fig. 14:**
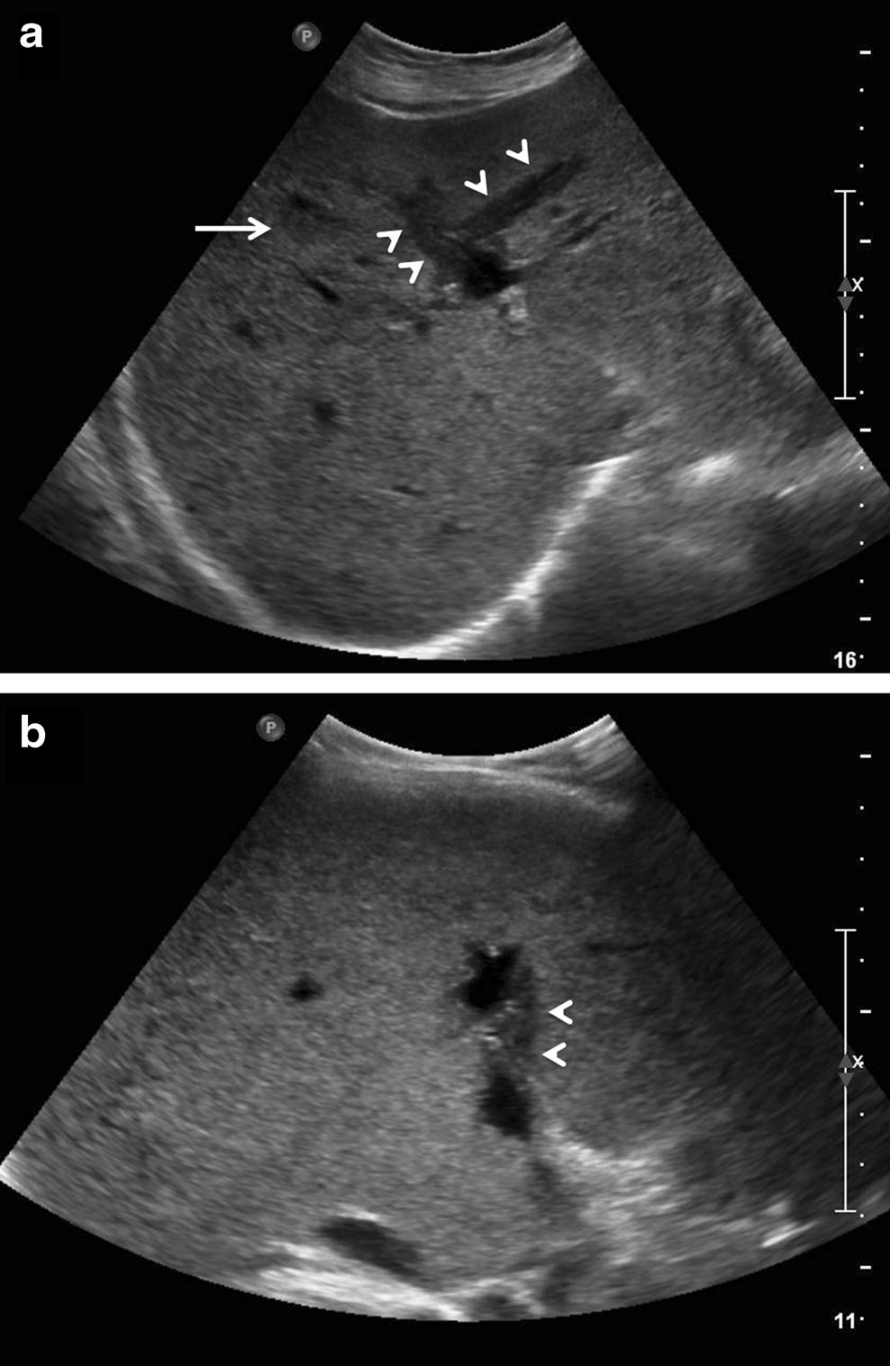
Non-Hodgkin lymphoma in a 34-year-old female with fulminant hepatic failure. Gray-scale US images demonstrating diffuse linear as well as nodular hypoechoic periportal cuffing (arrowheads and arrow, respectively). The patient had a rapidly downhill clinical course and succumbed to her illness within a week of admission. Post-mortem liver biopsy revealed findings consistent with diffuse large B-cell lymphoma
  More commonly, however, a large seemingly infiltrating juxta-hilar mass is seen tracking along the biliary radicals and portal tracts. On US, the mass is usually hypoechoic. However, areas of heterogeneity as well as completely echogenic lesions have also been reported [49]. On CT, these masses are homogeneously hypodense and show poor enhancement (Fig. 15). Also, as previously described, they encase the portal vessels but do not occlude them, connoting their pliable nature and soft texture. Owing to its better soft tissue contrast, MR imaging can delineate the entire extent of these tumours, particularly when combined with diffusion weighted imaging. The severity of biliary dilatation can also be assessed simultaneously (Fig. 16).

**Fig. 15:**
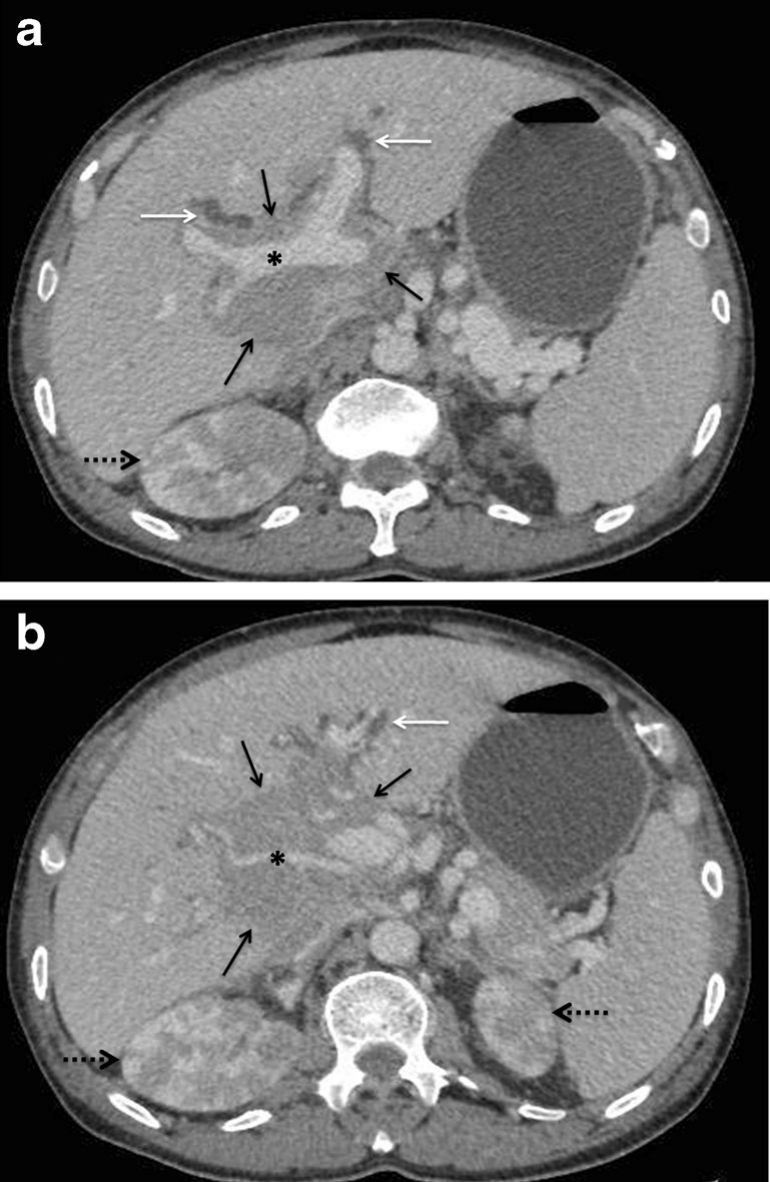
SHL in a 60-year-old man with jaundice. Axial CECT images demonstrating an ill-defined hypoenhancing periportal soft-tissue mass (solid black arrows in **a** and **b**) causing mild dilatation of the intrahepatic biliary radicals (white arrows). Portal vein and hepatic artery (asterisks in **a** and **b**, respectively) are seen coursing through the lesion without being attenuated or thrombosed. Bilateral kidneys are also involved (interrupted arrows)

**Fig. 16:**
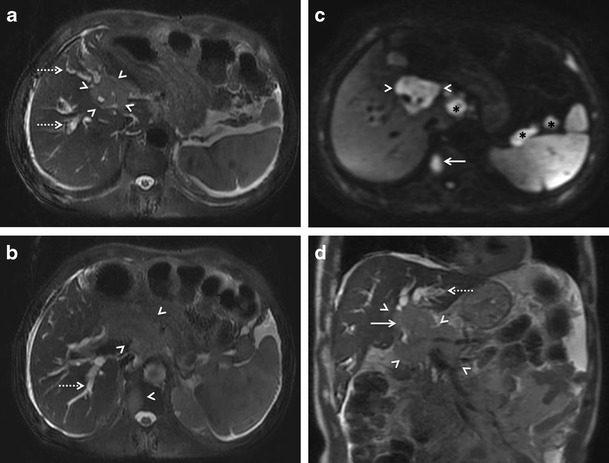
MRI findings in periportal infiltrating variety of hepatic lymphoma in a 60-year-old man. Axial (**a** and **b**) and coronal (**d**) T2-weighted and diffusion weighted (**c**) MR images demonstrating a mildly hyperintense ill-defined juxta-hilar diffusion restricting mass (arrowheads) completely encasing the common bile duct (solid arrow in **d**) and vessels at porta with resultant upstream biliary dilatation (interrupted arrows). A vertebral lesion is also seen (solid arrow in **b** and **c**) along with perihepatic and splenic hilar lymph nodes (asterisks in **c**)

## Staging and follow-up

Currently, prognostic indices are mostly used to risk-stratify patients and decide therapy, but most of these indices include stage as a factor, so imaging-determined stage remains relevant. A modification of the Ann-Arbor classification has been retained for this purpose in the Lugano criteria (Table 3) [38]. Treatment is practically based on limited (stages I and II, nonbulky) or advanced (stages III or IV) disease, with stage II bulky disease considered limited or advanced as determined by histology and a number of prognostic factors. The designation E is used for limited extranodal disease in the absence of nodal involvement (stage IE) or in patients with stage II disease and direct extension to a non-nodal site. E is not relevant to patients with advanced-stage disease. Therefore, accurate detection of hepatic involvement at initial presentation is important in staging. Lugano criteria formally include FDG-PET/CT as the standard imaging study for staging of FDG-avid lymphomas. CT scan is preferred in the other non- FDG-avid or variably FDG-avid lymphomas. A bone marrow biopsy has been excluded for the routine staging of HL and most diffuse large B-cell lymphomas.

**Table 3:** Revised staging system for primary nodal lymphomas*

Stage	Involvement	Extranodal (E) status
Limited		
Stage I	One node or a group of adjacent nodes	Single extranodal lesion without nodal involvement
Stage II	Two or more nodal groups on the same side of the diaphragm	Stage I or II by nodal extent with limited contiguous extranodal involvement
Stage II bulky	II as above with “bulky” disease	Not applicable
Advanced		
Stage III	Nodes on both sides of the diaphragm; nodes above the diaphragm with spleen involvement	Not applicable
Stage IV	Additional non-contiguous extralymphatic involvement	Not applicable

*Adapted from reference [38]

In a patient with known nodal disease, secondary liver involvement on FDG-PET/CT is usually indicated by visual assessment of diffusely increased or focal uptake, with or without focal or disseminated nodules. Presence of palpable liver with abnormal liver chemistries serves as a useful complementary clue. However, PHL is more difficult to characterise purely on imaging, and histopathological confirmation is usually required.

Imaging also plays a crucial role in the assessment of response to therapy. Early assessment of response to therapy allows more individualized therapy that will maximize the chance of cure while minimizing risk of toxicity. However, anatomic imaging modalities such as CT, US, and conventional MRI sequences may not be sufficiently accurate in discriminating residual disease from fibrosis or scar tissue. FDG-PET/CT, on the other hand, provides functional tissue information, and is superior to conventional imaging modalities in this context. It shows metabolic response earlier than anatomic response and has been recommended as the standard of care for response evaluation.

## Treatment

Secondary hepatic lymphoma is typically treated with chemotherapy, with the treatment regimen dictated by the histological subtype. In cases of PHL presenting as a solitary lesion or limited hepatic disease, surgical resection followed by a combination of chemotherapy and radiotherapy have also been tried, although no established treatment regimen exists. In general, PHL has a poor prognosis and the mean survival time following chemotherapy is 14 months. In contrast, the mean survival time following surgical treatment is 32 months, and localized PHL that can be completely resected has a relatively good prognosis leading some authors to recommend surgery as the first-line treatment in these cases [2, 50].

## Conclusion

There are no pathognomonic imaging features to diagnose hepatic lymphoma. However, presence of a homogeneous hypoenhancing intraparenchymal lesion with penetrating vessels or periportal soft-tissue lesion without any significant mass effect in a middle aged male without any known primary should alert one to the possibility of primary lymphomatous involvement of liver, particularly if there is a history of immunosuppression. Secondary involvement of liver by lymphoma is relatively easier to diagnose on imaging due to the concomitant involvement of other organs especially spleen and generalized lymphadenopathy, although differentiation with disseminated granulomatous infection may sometimes be difficult.
